# Errata: EPI-DRC Brasil: triagem da doença renal crônica com teste rápido de creatinina durante o Dia Mundial do Rim

**DOI:** 10.1590/2175-8239-JBN-2025-0298erpt

**Published:** 2026-06-26

No artigo “EPI-DRC Brasil: triagem da doença renal crônica com teste rápido de creatinina durante o Dia Mundial do Rim”, com o DOI https://doi.org/10.1590/2175-8239-JBN-2025-0298pt, publicado no periódico Brazilian Journal of Nephrology, 48(3):e20250298, 2026, na página 7:

Onde se lia:

**Figure:**
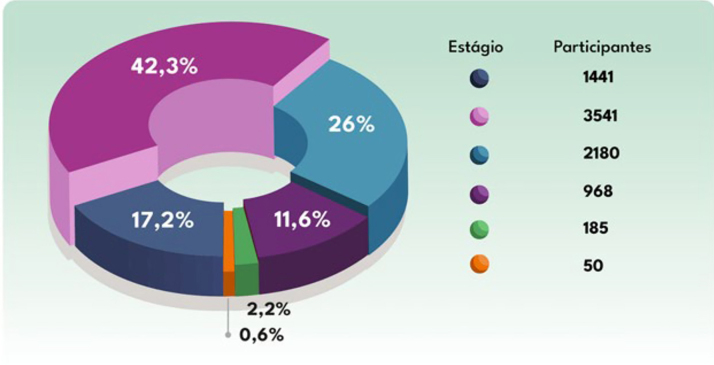


Leia-se:

**Figure:**
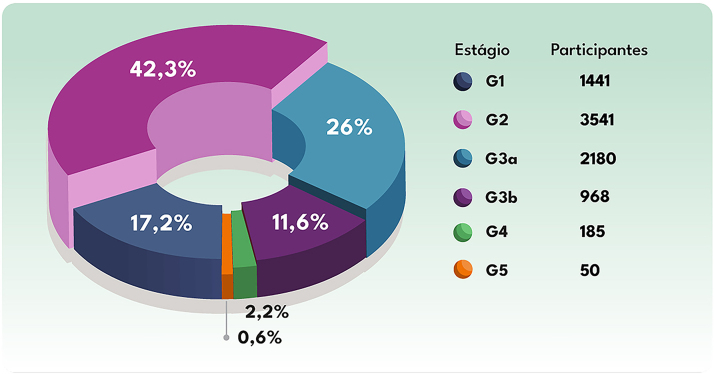


**Responsabilidade Editorial**

Editor-chefe: Miguel C. Riella https://orcid.org/0000-0003-4181-613X.

